# Engaging perioperative students in online learning: Human factors

**DOI:** 10.1177/17504589221107227

**Published:** 2022-09-03

**Authors:** Sinead Mehigan, Anne Slyne Cenarosa, Roslyn Smith, Marionette Zvavamwe, Michael Traynor

**Affiliations:** 1 Middlesex University London, London, UK; 2 Barts Health NHS Trust, London, UK; 3 Maternity Unit, Whittington Health, London, UK; 4 Acute Assessments, SDEC & Ambulatory Emergency Care Unit, Acute Medicine, Perioperative, Critical Care & Emergency Care Division, Barking, Havering and Redbridge University Hospitals NHS Trust, Romford, UK

**Keywords:** Online learning, Human factors, Student engagement, Communities of inquiry, Simulated learning, Reflection, Debriefing

## Abstract

The aim of this article is to reflect on how a specific approach to teaching and learning – role-play and in particular the ‘radio play’ format – can overcome some of the alienation and barriers to student engagement that the necessity for online teaching can engender. The example used is a post-registration module in perioperative nursing delivered in a London University. Authors reflect on experiences of developing and implementing an activity designed to increase student engagement in an online session focussing on human factors in the perioperative setting. The aim of the session was to highlight the factors that potentially lead to clinical error in a way that engaged students and enabled them to relate learning to their own experiences. The challenge was how best to do this in an online setting. This article describes the use of an approach devised by AdvanceHE. Two student participants contribute their reflections to this article and focus on the extent to which the session facilitated a new understanding of the impact of human factors in a perioperative setting. These reflections suggest that the approach to the teaching employed was useful to participants and that it has promise as an online approach. The authors recommend evaluation of this approach.

## Introduction

With access to face-to-face teaching during the pandemic reduced, educational opportunities for many perioperative staff moved online. This article focuses on the experience of delivering one session on a short post-registration perioperative nursing module in a London University online. Normally, the five-day 15-credit module is delivered face-to-face. The module forms part of our Continuing Professional Development portfolio and can contribute to a range of postregistration academic awards. It is generally accessed on a stand-alone basis by perioperative staff with some experience of practice, who want to further develop their knowledge base and clinical decision-making. We use simulated learning scenarios in some sessions to help students explore aspects of practice and consider how they might improve their clinical decision-making. Such sessions include one on caring for the deteriorating patient, and the particular session discussed in this article – human factors. The challenge for the academic team was on how to translate face-to-face learning experiences to an online setting in such a way that students could engage with each other and with the learning materials. During this time in 2020, when there was a need for academics to adapt provision to finding more flexible forms of teaching delivery, AdvanceHE produced a toolkit which contained 52 practical, but high-impact activities, which they designed to increase student engagement (Turner et al 2020). In this article, two students and two academics reflect on their experiences of using one of these activities in a two-hour online teaching session, which focused on the topic of human factors in clinical practice. The structure used to reflect on the session is loosely based on the work of Driscoll (2007) and Gibbs (1988). The process of reflection has given the writing team the opportunity to consider broader lessons in facilitating and debriefing simulations in an online setting.

## Human factors in perioperative practice

The operating room (OR) is identified as a high-risk environment with increased risk of adverse events occurring (de Vries et al 2008). Despite the introduction of numerous patient safety initiatives, including the ‘Five Steps to Safer Surgery’ (2010) from the National Patient Safety Agency (NPSA 2010), the incidence of surgical Never Events in the National Health Service (NHS) is still considered unacceptably high (NHS England and NHS Improvement 2019). Never Events are defined as ‘... Serious Incidents that are wholly preventable because guidance or safety recommendations that provide strong systemic protective barriers are available at a national level and should have been implemented by all healthcare providers’ (NHS Improvement 2018: p4). Many analyses of Never Events within the perioperative setting have focused on human factors that contribute to their occurrence (Omar et al 2020). Human factors refer to ‘environmental, organisational and job factors, and human and individual characteristics, which influence behaviour at work in a way which can affect health and safety’ (HSE 2009: p5). Within the perioperative setting, this includes the impact of stress and fatigue, or failures in communication between team members, time pressures or the influence of the medical hierarchy on staff performance (Roche 2016).

## Student engagement – moving from classroom to online settings

Prior to the pandemic, we had created a series of learning activities, designed to better engage students through active learning in a classroom setting. Engagement, in this context, refers to student participation in purposeful learning activities, which have been designed to improve student attention, interest in a subject and achievement of learning outcomes (Evans et al 2015). Feedback from students on our initial attempts of running online sessions in 2020 suggested that they struggled to engage with learning in an online teaching session. In an effort to address this feedback, we drew on the toolkit developed by AdvanceHE, called #52etc (Turner et al 2020), to re-design a number of sessions. The toolkit is presented as a series of playing cards, each giving a low-tech practical activity designed to enhance student engagement, whether in a classroom, online or in blended learning. All activities in the toolkit have been designed to help increase student involvement in their studies and to motivate and engage them. The activities have also been designed using key principles underpinning teaching and learning (Evans et al 2015) including the use, through simulation, of real-world examples, and using experiential approaches to enhance student understanding.

## Detail of the session

Fourteen students took part in a two-hour online session. We adapted one of the activities – from a set of 52 – produced by Turner et al (2020) as described above, in an effort to increase student engagement and learning about human factors within the perioperative setting. The activity selected suggested ways to encourage students to reconsider an issue from an alternative perspective – with the option of using role-play. We asked students prior to the session, if they would be willing to create and perform in a radio play. We chose this as a form of simulation well suited to students working remotely online, because in some respects, it mimics the experience of listening to a radio broadcast. We chose to understand the performance as a radio rather than television play because the presentation was largely speech rather than action-based. We felt this might be effective in giving them the opportunity to test communication and critical thinking skills, by tapping into their creativity by having to project their thoughts, emotions into a ‘role,’ while sitting at their desk at home. Five students agreed to write and perform the play.

One of the teaching team developed a draft script to guide students in creating their play; however, we wanted to ensure that participants were able to improvise. We felt that giving students the opportunity to shape the material was key to the transformational learning experience for both student actors and the remaining nine students watching as this gave the opportunity for learning by imaginatively entering into the material, and the use of self-reflective learning for all participants.

At the beginning of the session, the ‘actors’ went into an online breakout room with a teacher to create their radio play, aimed at highlighting some of the factors that can contribute to mistakes happening in the perioperative setting. Given that we did not have large groups of students to take on all roles, we gave some actors a number of different roles to play. We had to think of a way that these actors could be identified in their different roles, so we used labels that could be shown on-screen and hats that could be worn as participants turned on their cameras to take on different roles. This took the group one hour. The remaining students were broken into small groups of three, and put into other breakout rooms to read a variety of articles (Koleva 2020, Reason 2000, Widdecombe & Owen 2017) which focus on human factors within the perioperative setting. The play, when performed, lasted for about ten minutes. A subsequent discussion regarding both the events that were represented in the play and the learning experiences of both actors and student observers was undertaken at the end of the play to consolidate learning. Following this session, we asked if any student would be interested in helping write a reflective article at a later stage. Two students volunteered to do so. In the following sections, these students, both of whom took part in the play as actors, contribute their reflections on this experience. Their accounts give evidence of the insights that an imaginative engagement with a familiar scenario can give rise to. As this initiative was a service evaluation, ethical approval was considered to be not needed.

## Student reflection – what was I thinking or feeling, as I took part in this play?

*In this activity, I was allocated to play the role of an inattentive Consultant Anaesthetist. I am a scrub nurse by profession and what I portrayed was purely based on what I thought would be fit for the character. I participated in the play and was given feedback that I portrayed the role well. The character weighed in on me because my profession as a scrub nurse is the complete opposite of [the one I played in the play]. I was trained to pay attention to the procedure, the instruments, the needs of the surgeon and most especially to be an advocate of the patient. In the activity, I did things like unnecessarily distracting the ODP during a Sign In because my role had to not pay attention to the stages of preoperative check.* (Student 1)

*It was quite daunting at first because I was very self-conscious and wanted to do a good job on something which was improvised and have a limited time to plan. Plus, it did not give me the ability to hide in the back of the virtual classroom . . . Once we started talking through how we were going to engage with each other during the play it became quite fun. It helped you to engage that childlike imagination that you do not get to visit within your perioperative role as things are structured with protocols and flow charts. Once we started the role play, it went better than I thought, and I think as a group we were able to put ourselves in the shoes of our characters and draw on experiences we have had and use that for inspiration and context. For me, this is what I used to draw on for the play. I enjoyed it.* (Student 2)

## So what? Making sense of this experience

As facilitators of the session it was clear to us, while students performed their play, that they had engaged with the process of creating and performing it, and how much energy the enjoyment of performance seemed to engender. It is also possible that this type of novel enjoyment and engagement helped with their learning, by tapping into their creativity in ways they might not have been able to do in practice or in more formal learning settings. For Student 2, their perception was that learning felt more active and that the experience led to deeper learning than if passively listening and taking notes from a lecture. This is something that is worth exploring in a future evaluation.

One of the interesting outcomes of this session was feedback from some of the actors – particularly those playing distracted or frequently absent members of staff, that is, staff who were performing suboptimally and perhaps dangerously. One student expressed that although they were playing a role, they felt uncomfortable doing so, as what they portrayed did not reflect their own normal practice. We understood this discomfort engendered by the role-play not only as evidence of the power of this approach to learning that engages the emotions but also as a reminder of the need to debrief with participants after such work.

## Student reflection – what have I learnt from this experience?

*The activity taught me the importance of understanding human errors in the surgical environment [that like other safety critical industries, we should accept that human errors are] inevitable and occur in our daily life (Bromiley & Mitchell 2009). Moreover, I have two things to take home from the activity: situational awareness and being a part of the surgical team with clear defined role. I feel that these are important non-technical skills to promote patient safety.* (Student 1)

*Being part of the play helped twofold because I was a character as well as an observer because I had to observe and react to what was being said while maintaining the context of what we were trying to portray. I think this helped me think deeper about human factors because within my part I wanted to make sure the audience understood what was going on. It was fun, using my imagination in this way was a pleasure rather than the conventional method of learning with someone talking to you, you listen and write notes. It helped with deeper learning and helped with my understanding of how human factors can play a big role when things go wrong due to miscommunication, assumptions, speaking up, power play within the perioperative environment, etc. It also helps with having to engage with the learning material, I had to and had no choice in a creative way.* (Student 2)

Although the accounts above suggest that the actors learnt from this experience in ways that they felt they would not have during ‘conventional’ teaching, it is important also to consider the degree of engagement and learning for those observing. During our debriefing, discussions among the whole group indicated that students observing were able to identify key areas where errors may occur. They were also able to draw on the literature they had read while in their own breakout areas on human factors. Some drew on learning from a previous session, where they had looked at caring for the deteriorating patient, to lead a wider discussion on what should have happened. General discussion after the performance also showed how students were able to identify with elements of the play, reflecting on how these related to incidences within their own practice. In evaluating the session, all students made clear links between the session and how they might apply learning to future practice.

## What knowledge can be applied from theory or research?

In exploring the effectiveness of this session, the Community of Inquiry (CoI) model helps to provide a useful lens for examining online learning processes (Garrison & Arbaugh 2007). Originally developed in 2000 (Garrison et al 2000) for asynchronous, text-based online learning environments, it has subsequently been applied to synchronous online learning (Cheng et al 2020). The framework describes three interrelated elements that contribute to the student’s overall learning experience. These are social presence, teaching presence and cognitive presence. *Social presence* involves creating a climate where students have built a sense of belonging and commitment to learning. This session was on one of the last days of the module, after students had worked together on a wide range of learning activities. This contributed to the feeling of mutual trust in the group. *Teaching presence* relates to the way in which the teaching team structured and set up the session, facilitated discussion, debriefed and summarised key learning, which for this session, related to human factors. We had briefed the students before the session of what we intended to do and asked them to consider taking roles as actors. We had previous experience of running simulations, in face-to-face sessions, based around human factors. *Cognitive presence* can be seen as the extent to which learners critically reflect and construct meaning on a topic (Cheng et al 2020). In this session, this was evident through the exchange of ideas and discussion that emerged following performance of the play. The learning from this process is also seen in the student reflections as quoted earlier in this article. We would suggest that students’ willingness to engage with the play and share ideas is closely linked to the fact that they were a cohesive group, with good experience of working in a common area of practice – perioperative nursing. There were also only 14 in the group, which may have helped enable the development of group cohesion in an online learning setting.

As stated, the aim of the session was to provide students with an opportunity to reflect on the impact of human factors on patient safety in the perioperative environment, but to do so in a ‘safe’ learning environment. Benner (2015) underlines the importance of providing situated learning experiences for students. Using the construct of a ‘radio play’ created by the students to a brief from the teaching team, seems to have offered the group exposure to a clinical scenario similar to ones they had come across in practice, which, although simulated, nevertheless provided a useful learning experience. Many of the students – both actors and observers – recognised and were able to reflect on the behaviours of those in the play, for example, an over-confident and talkative healthcare support worker, or a distracted scrub nurse. The discussion after the play enabled them to share ideas in a safe environment of how they might respond to such behaviours differently in the future by being more assertive. Working with those students who created the play gave us, as a teaching team, a sense of partnership working on their learning. For the students contributing reflections to this article, what emerges is a sense of belonging and engagement with that learning. Turner et al (2020) give examples of principles, or ways of working that educators might adopt to underpin inclusive and supportive engagement. These include respecting the diversity of experiences and identities of all involved in this learning experience, and on reflecting in a way that is informed by our connections to the learning materials. The fact that the play was created by the students, helped ensure that we were able to draw on their different experiences and identities in the virtual room.

For some of the actors taking on other identities helped them to view practice from another perspective, and to reconsider their own values and practices. This session also helped students develop further awareness of the importance of non-technical skills, which, along with technical expertise, help ensure that members of the perioperative team work together to develop safe and effective practice (Mitchell & Flin 2008, Sevdalis 2013).

One issue arising during the debriefing was that playing a role, for some, felt uncomfortable. Matthews et al (2019) stress the need to consider the challenges of creating safe discussion, and to be aware of the influence of power imbalances between teacher and learner, whether overt or hidden. This session has led the academic team to consider methods of effective debriefing in an online setting. It can be more difficult, particularly in terms of creating a safe space for discussion (Turner et al 2020). It is more difficult to pick up cues of discomfort from looking at faces on a screen.

## Conclusion

The aim of this article was to reflect on how a specific approach to teaching and learning – role-play and in particular the ‘radio play’ format – appears to overcome some of the alienation and barriers to student engagement that the necessity for online teaching can engender. The session used as an example in this article focused on exploring the impact that human factors can have on patient safety within a perioperative setting. The creation of an online ‘radio play’ by the students, which simulated a possible clinical scenario was inspired by one of the suggested activities from #52etc student engagement toolkit (Turner et al 2020). Feedback from students suggests that the activity did engage them more than ‘conventional’ teaching might – and helped them reflect on and develop a greater understanding, not only about human factors but also of the importance of non-technical skills in their roles within the perioperative setting. The toolkit was designed with some of the challenges of providing learning in an online setting in mind.

Student feedback, both formal and informal, suggests that the activities have potential to promote dialogue beyond the classroom space (Turner et al 2020). Although formal evaluations are needed to identify the benefits of any educational initiative, it appeared that the engagement engendered by the session was, perhaps, due in part to the social presence of students (Cheng et al 2020, Garrison et al 2000). Key to this was working with a small group of students who had already been working together for some time on a module. It would be interesting to see if this approach could be replicated with larger groups of students.
